# Comprehensive assessment of factors contributing to the actual turnover of newly licensed registered nurses working in acute care hospitals: a systematic review

**DOI:** 10.1186/s12912-023-01190-3

**Published:** 2023-02-04

**Authors:** Sung-Heui Bae

**Affiliations:** College of Nursing, Graduate Program in System Health Science and Engineering, Ewha Womans University, Helen Hall #204, 52, Ewhayeodae-gil, Seodaemun-gu, Seoul, 03760 Republic of Korea

**Keywords:** Newly licensed registered nurses, Turnover, Acute care hospitals, Systematic review

## Abstract

**Background:**

During the COVID-19 pandemic, the demand for nursing care increased, making the retention of nurses even more important. Among staff nurses, it is reported that the turnover rate of newly licensed registered nurses is higher. However, no systematic reviews have focused on the factors that influence newly licensed registered nurses’ turnover. Additionally, because newly licensed registered nurses are a major source of the supply of nurses, it is critical to retain them to meet patient needs. Therefore, this study aimed to systematically synthesize the factors contributing to the actual turnover of newly licensed registered nurses working in acute care hospitals.

**Methods:**

CINAHL, Cochrane Library, DBpia, EBSCO, PubMed, PsycINFO, RISS, and Web of Science were searched for studies published between January 2000 and June 2021. This systematic review followed the Preferred Reporting Items for Systematic Reviews and Meta-Analyses guidelines.

**Results:**

Ten articles from 9029 were included in this review. All studies used a longitudinal design. The annual turnover rates of newly licensed registered nurses ranged from 12 to 25%. Health status, including sleep and healthy lifestyles, were significant factors affecting turnover. Most studies focused on work environment factors, and emotional exhaustion, job satisfaction, peer support, and intent to leave, were significantly associated with newly licensed registered nurses’ turnover. Small hospitals located in nonmetropolitan areas were at risk of high turnover of newly licensed registered nurses.

**Conclusions:**

Turnover is inevitable in the process of employment, but high turnover can be prevented. Through reviewing ten articles, significant contributing factors for newly licensed registered nurses’ turnover included personal factors of health status; work environment factors of physical exhaustion, emotional exhaustion, depersonalization, occupational injuries, income, intent to stay, job satisfaction, and peer support; and hospital factors of hospital size, location, and unionization. Most existing studies focus on work environment factors, which reflects the significance of fostering healthy work conditions to prevent high turnover. These findings can be used to develop strategies and policies for work environment to reduce high turnover of newly licensed registered nurses, and support high-risk groups, such as small hospitals located in nonmetropolitan areas with high levels of nurses’ turnover.

## Background

The projected shortfall of nurses is expected to reach 10 million by 2030 [1]. To respond to such nursing shortages, the World Health Organization (WHO), the International Council of Nurses (ICN), and Nursing Now strongly recommend that governments and stakeholders substantially invest in nursing education, jobs, and leadership for the nursing workforce [2]. Given the nursing shortages, nurses’ high turnover is an international concern [3]. During the COVID-19 pandemic, nurses experienced anxiety at work, the fear of infection, elevated workloads, shifts without sufficient rest, and high patient-nurse ratios, which increased organizational and professional turnover intentions among nurses [4–6]. Nurse turnover rates were reported to be 27.65% in the USA [7], 23% in Israel [8], and 12.4% in South Korea [9].

Nurse turnover had detrimental effects on nurse and patient outcomes. An increase in annual turnover rates among registered nurses (RNs) is related to the physical and mental health of nursing staff [10]. In terms of patient outcomes and quality of care, nurse turnover rates were negatively related to patient satisfaction [11]. Unit-acquired pressure ulcers and medical errors increased when RN turnover increased [12]. In another study, however, nurse turnover was not found to be related to patient outcomes [13]. Nurse turnover is also considered to be very costly and consists of pre- and post-hire costs, which include temporary replacement costs and decreased productivity of newly hired nurses [14]. The cost per turnover is estimated to be 3 times that of a nurse’s salary [14].

Among staff nurses, the turnover of newly licensed registered nurses (NLRNs) is reported to be even higher. For example, in South Korea, the turnover rate of NLRNs was 42.7% in 2017 [9]. In the US, their turnover rate is considerably higher than that of experienced nurses [15]. A substantial proportion of NLRNs start their careers in hospitals, where they are a major human resource [16]. When nurses leave their first hospital jobs, they are less likely to continue working in acute care areas [17]. Thus, it is crucial to identify factors that contribute to NLRNs’ turnover and accordingly develop strategies to retain them.

Several reviews have recently been conducted to summarize and synthesize factors that contribute to actual turnover among nurses. Halter et al. [18] examined nine systematic reviews to identify factors contributing to turnover among nurses who provide nursing care to adult patients and found that nurse work-related stress and dissatisfaction at the individual level and managerial style and supervisory support at the organizational level were important factors. Falatah and Salem [19] appraised literature on nurse turnover in the Kingdom of Saudi Arabia. They included studies examining both nurse turnover and turnover intention and found the following determinants: nurses’ demographics, satisfaction, leadership and management, and job-related factors. McDermid et al. [20] reviewed 20 articles to synthesize the factors contributing to high turnover rates of nurses working in emergency departments and found three major factors, including aggression and violence, critical incidents, and work environment.

However, no systematic reviews focus on factors affecting NLRNs’ turnover. NLRNs are a major source of supply of nurses [21]; thus, it is important to retain them to meet patient needs. As mentioned before, their turnover rates are higher than those of experienced nurses [15], indicating the necessity of understanding the factors affecting these nurses’ turnover. Therefore, this review appraised and synthesized studies examining NLRNs’ turnover and factors contributing to it. In this review, nurse turnover is considered the actual turnover of NLRNs, not intent to leave or turnover intention as it may not lead to actual turnover [22]. Price’s [23] conceptual framework of turnover includes personal characteristics, work attitudes and conditions, and job opportunities. In this review, the contributing factors were categorized into personal, work environment, nursing unit, hospital, and community factors. Work environment factors were synthesized based on Price’s turnover model [16, 23]. This study’s findings can be used to develop programs, strategies, and health policies to prevent and reduce NLRNs’ turnover.

### Aims

This systematic review aimed to investigate the factors contributing to NLRNs’ turnover in acute care hospitals and synthesize evidence regarding these contributing factors at the personal, work environment, nursing unit, hospital, and community levels.

## Methods

### Search methods

The Preferred Reporting Items for Systematic Reviews and Meta-Analyses guidelines [24] were used to report this review. Eight electronic bibliographic databases—CINAHL, Cochrane Library, DBpia, EBSCO, PubMed, PsycINFO, RISS, and Web of Science—were used to identify relevant articles. The following search terms were used: (a) “nurse” AND “turnover” AND “acute,” (b) “nurse” AND “turnover” AND “hospital,” (c) “nursing” AND “turnover” AND “acute,” and (d) “nursing” AND “turnover” AND “hospital.” Abstracts, titles, keywords, author keywords, keyword plus, and MeSH terms were searched to get the relevant studies. To include all relevant articles, the search terms did not include study population (e.g., NLRNs). Through the review process, only articles studying NLRNs were included. Similarly, independent variables (contributing factors) were not specified in the search terms to identify all relevant articles regardless of the type of independent variables. The search was conducted in June 2021. Two reviewers independently identified relevant articles. Approval from an institutional review board was not required because this review did not involve data collection from human participants.

Articles were selected based on the following eligibility criteria. They: (1) investigated factors affecting actual organizational turnover of NLRNs, (2) were original research published in peer-reviewed journals, (3) were non-experimental quantitative studies, (4) were written either in English or Korean, and (5) were published between January 2000 and June 2021.

### Search outcomes

**Fig. 1:**
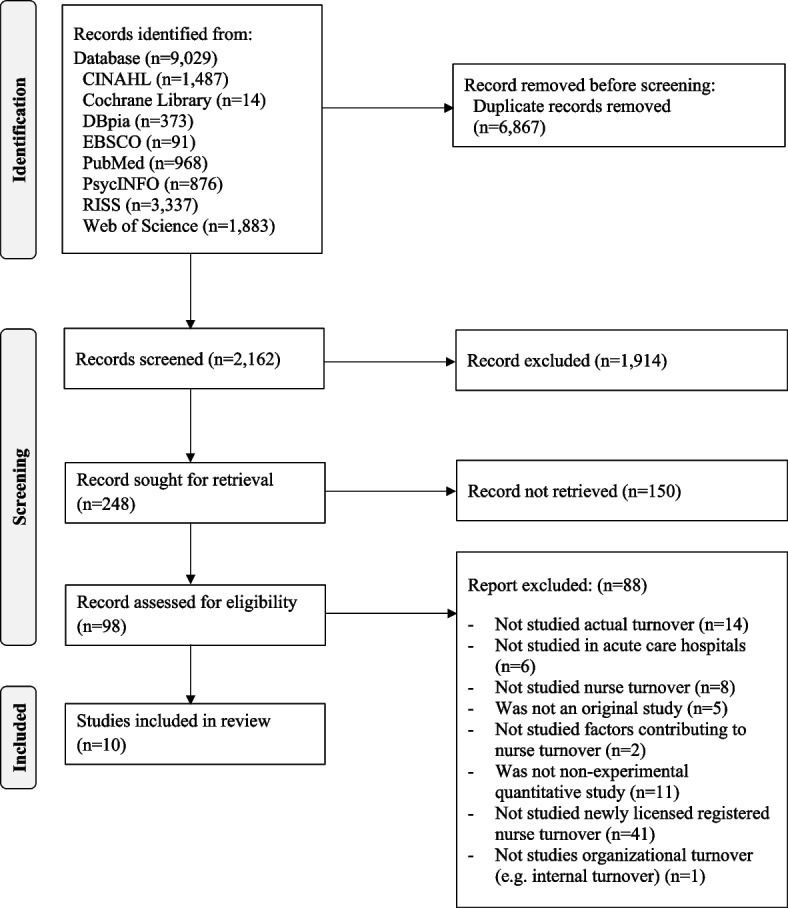
PRISMA flowchart of systematic review

Of the 9029 articles retrieved from eight databases, 6867 duplicates were found and removed, leaving 2162 articles (Fig. 1). Based on the inclusion criteria, the titles were screened and 1914 were excluded. Of the 248 remaining articles, 150 were excluded after screening the abstracts. Full text screening resulted in the exclusion of 88 articles from the remaining 98. Articles were excluded if they: (a) did not examine actual turnover (e.g., examined intent to leave) (*n* = 14); (b) did not conduct a study in acute care hospitals (e.g., long-term care organization) (*n* = 6); (c) did not examine nurse turnover (*n* = 8); (d) were not original studies (*n* = 5); (e) did not examine factors contributing to nurses’ actual turnover (*n* = 2); (f) were not non-experimental quantitative studies (*n* = 11); (g) did not examine NLRNs (*n* = 41); and (h) did not examine organizational turnover (e.g. internal turnover) (*n* = 1). Finally, ten articles were included in this review, for which methodological quality assessment was performed.

### Quality appraisal

Table 1 presents the items for quality appraisal of the included studies and the number of articles corresponding to each item. The quality assessment tool of 13 items comprised of design (one item), sample (five items), measurement (four items), and statistical analysis (three items) was adopted from previous studies [25, 26]. Items in the measure were modified according to the research question. Scores were calculated based on the number of “Yes” responses for each item; the total score ranged from 0 to 13. Higher scores indicated better study quality. The total score of each study was categorized into low (less than four), medium (between five-nine), and high (greater than ten).

**Table 1:** Summary of quality assessment of the studies included

	Brewer et al. (2012) [16]	Cho et al. (2012) [21]	Halfer (2011) [27]	Han et al. (2019) [17]	Han et al. (2020) [28]	Kim & Lee (2016) [29]	Lee (2019) [22]	Suzuki et al. (2006) [30]	Suzuki et al. (2008) [31]	Suzuki et al. (2010) [32]	Total (Yes)
Design											
Was the study other than cross-sectional (longitudinal study)?	1	1	1	1	1	1	1	1	1	1	10
Sample											
Was probability sampling used?	1	1	0	0	0	1	1	0	0	0	4
Was the sample size justified?	1	0	0	0	0	0	0	0	0	0	1
Was the sample drawn from more than one site?	1	1	0	0	0	1	1	1	1	1	7
Was anonymity protected?	1	1	1	1	1	1	1	1	1	1	10
Was the response rate more than 60%?	0	0	1	1	1	0	0	1	1	1	6
Measurement											
Were the independent variable measured reliably?	1	1	1	1	1	1	1	1	1	1	10
Were the independent variable measured using a valid instrument?	1	1	1	1	1	1	1	1	1	1	10
Was the nurse turnover variable measured reliably?	1	1	1	1	1	1	1	1	1	1	10
Was a theoretical model/framework used for guidance?	1	1	0	0	0	0	0	0	0	0	2
Statistical analysis											
If multiple effects were studied, were correlations analyzed?	1	0	0	0	0	0	0	0	0	0	1
Were covariates controlled?	1	1	1	1	1	1	1	1	1	1	10
Were outliers managed?	0	0	0	0	0	0	0	0	0	0	0
Total	11	9	7	7	7	8	8	8	8	8	
Quality	High	Medium	Medium	Medium	Medium	Medium	Medium	Medium	Medium	Medium	

Cummings et al. [25] and Pennonek et al. [26] were used for quality assessment

### Data extraction and synthesis

To summarize and synthesize the review results, the following data were extracted (Table 2): author name, publication year, country, study design, sample, data collection time, measures and mean values of nurse turnover, measures of factors affecting nurse turnover, analysis methods, quality score and category, and main findings regarding the significant factors that contribute to turnover among nurses.

**Table 2:** Characteristics of studies

#	Authors (year), country	Study design/ sample/data collection time	Measures/mean values of nurse turnover	Measures of factors	Data analysis/ quality score/quality	Main findings (*p* < .05)
1	Brewer et al. (2012) [16], US	Longitudinal panel design/ 1653 RNs/ 2006, 2007	Whether at time two the NLRNs had stayed or left their employers identified at time one/14.5% (external turnover rate), 13.3% (internal turnover rate)	Personal characteristics, work attributes, opportunity, shocks, work attitudes [33, 34]	Binomial probit regression/ 11/high	**-** Work attributes (more than one job, working full time, voluntary overtime), shocks (sprains or strains), and work attitudes (intent to stay) influenced turnover. - Non-significant relationships between personal characteristics (age, gender, ethnicity, first language, marital status, children less than 6 years old, overall health, positive/negative affectivity, spousal income, income from other sources, first nursing degree, times took NCLEX), work attributes (staff/general duty, first RN job: formal orientation, first RN job: reduced workload, imputed wage, RN benefits-formal education, total number of RN benefits, number of months worked in current job, number of shifts floating, previous work experience, externship, internship, Magnet hospital, mandatory overtime, patient load, type of shift, typical work schedule, unit type, number of times change in supervisor, part of a union, importance of benefits from RN job), opportunity (local/non-local job opportunity, percent of all persons in HMOs, beds per 1000 population, unemployment rate, MSA), shocks (needle sticks, verbal abuse), work attitudes (job search behavior, job satisfaction, organizational commitment, variety, autonomy, mentor support, supervisory support, workgroup cohesion, distributive justice, procedural justice, promotional opportunities, work motivation, quantitative workload, organizational constraints, collegial RN-MD relations, work family conflict, family work conflict) and nurse turnover were found.
2	Cho et al. (2012) [21], South Korea	Longitudinal design/ 351 RNs/ 2006, 2007, 2008	RN-reported turnover/45% (before the 3rd year)	Age, gender, marital status, father’s education, nursing education, hospital size, location, presence of labor unions, job satisfaction	Cox proportional hazards regression analysis/ 9/medium	- Married RNs working in small, nonmetropolitan areas, nonunionized hospitals reported higher turnover. Several aspects of job dissatisfaction (work content, physical work environment, interpersonal relationships) were significantly associated with turnover. - Non-significant relationships between age, gender, father with 4+ years college, nursing education (nursing degree, reason for choosing nursing major), job dissatisfaction (working hours, social insurances and fringe benefits, advancement system, social reputation for the job) and nurse turnover were found in univariate analysis.
3	Halfer (2011) [27], US	Descriptive longitudinal design/ 116 RNs/2008, 2009	Whether the new graduate nurses stayed or left the organization between 2008 and 2009/12% (within a year)	Personal characteristics, job embeddedness factors [35]	Logistic regression modeling/ 6/medium	- Nurses who reported higher turnover were younger and had significantly lower agreement on two items of organizational embeddedness, which are: “I feel part of my work team” and “I feel as if I am a good match for this hospital”. - Non-significant relationships between personal characteristics (gender, marital status, number of children living at home, organizational tenure, education, ethnicity, part-time or full-time status, length of commute, whether nursing was a second career, and current position), job embeddedness (community embeddedness, organizational and community embeddedness item summed scores) and nurse turnover were found.
4	Han et al. (2019) [17], South Korea	Descriptive and prospective longitudinal study design/ 464 RNs/the first day of orientation before ward placement (between September 2014 and December 2015), 6 weeks after starting work, and at the 6th, 12th, 18th, and 24th month of work	Whether the nurse participants had stayed on or resigned by December 31, 2017/not reported	Pre-employment health lifestyle variables: sleep [36], diet behaviors, physical activity (Korean International Physical Activity Questionnaire Short Form [37, 38]), alcohol consumption [39], depressive symptoms (the Korean Center for Epidemiologic Studies-Depression scale [40, 41];), and self-rated health [42] , job stress (demands, control, and support)	Cox proportional hazards regression/ 7/medium	- Compared with the new nurses in the discordant group (low levels of sleep disturbance, depression, and poor self-rated health but relatively high levels of poor diet and physical inactivity behaviors), those in the unhealthy lifestyle group (high levels of poor sleep quality, not eating 3 meals a day, irregular diet, depression, and poor self-rated health) had significantly higher probabilities of resigning. - Non-significant relationships between job stress (psychological demand, physical demand, control, boss support, peer support) and nurse turnover were found.
5	Han et al. (2020) [28], South Korea	Prospective longitudinal design/ 465 RNs/the first day of orientation before ward placement (baseline, T0, between September 2014 and December 2015), 6 weeks after starting work (T1), and at the 6th, 12th, 18th, and 24th month of work (T 2–5)	Whether the new nurses had left the hospital at each data collection point/ 2.6% (until T1), 9.2% (until T2), 12.3% (until T3), 18.7% (until T4) 23% (until T5)	Personal characteristics (age, gender, marital status, highest degree achieved, alcohol consumption, smoking), trajectories of sleep disturbance (General Sleep Disturbance Scale [43, 44]) classified into the high/low symptomatic group	Latent growth curve analysis/ 7/medium	- Turnover rates of the high symptomatic group of sleep disturbance trajectories were higher than those of the low symptomatic group, and sleep disturbance was more severe among leavers than among stayers in the high symptomatic group. - Nurse turnover was not different according to the personal characteristics (age, gender, marital status, highest degree achieved, alcohol consumption, smoking) in univariate analysis.
6	Kim & Lee (2016) [29], South Korea	Secondary data analysis with retrospective study/ 323 RNs/ 2010 GOMS (2011, 2013)	Whether the new nurses had left their first job/24.5% (within the 1st year), 39% (within the 2nd year), 47.4% (within the 3rd year), 51.7% (within the 4th year)	General characteristics, job-related characteristics, satisfaction, characteristics of region	Multilevel survival analysis/ 8/medium	- Job-related characteristics (job status, monthly income), satisfaction (physical work environments, work hours, interpersonal relationship, social insurance and fringe benefits), and characteristics of region (number of hospitals in region, number of nurses per 100 beds) were significant predictors of turnover among new nurses. - Non-significant relationships between general characteristics (gender, age), job-related characteristics (number of employees), satisfaction (pay or income, stability of employment, potential for personal growth, advancement system, social reputation for the job, autonomy and authority for the job), characteristics of region (number of beds per 1000 population) and nurse turnover were found.
7	Lee (2019) [22], South Korea	Longitudinal panel design/ 652 RNs/ 2008 GOMS (2009, 2011), 2009 GOMS (2010, 2012) 2010 GOMS (2011, 2013)	Whether newly graduated nurses transferred profession or organization/25% (within the 1st year), 50% (within the 4th year)	Individual factors, organizational factors, job satisfaction	Cox regression analysis/ 8/medium	- Individual factors (graduation year, gender), hospital factors (interaction of hospital size and monthly salary, presence of union), dissatisfaction with organization and profession were major factors that affected the turnover rate. - Non-significant relationships between individual factors (gender, marital status, educational level of father, family income, admission type, selection reason), hospital factors (hospital location, moving direction for working, shift), job dissatisfaction (salary, welfare benefit, performance appraisal) and nurse turnover were found in univariate analysis.
8	Suzuki et al. (2006) [30], Japan	Longitudinal design/ 923 RNs/ 2003 (June), 2003 (December)	Turnover between novice nurses who quit their jobs between June 2003 to December 2003/4%	Nurse attributes, clinical department, burnout (the Japanese version of the MBI [45, 46]), assertiveness (the Japanese version of RAS [47, 48]), stressful life events (Social Readjustment Rating Scale [49]), reality shock, ward assignment preference, transfer preference, job satisfaction, social support and coping mechanisms [50]	Multiple logistic regression with the stepwise variable selection method/ 8/medium	- Graduation from vocational nursing school, dissatisfaction about being assigned to a different ward, and lack of social support from peers were the factors affecting rapid turnover. - Non-significant relationships between nurse attributes (gender, age, living arrangements, location of the hospital, nursing arrangement, ward, department), burnout (emotional exhaustion and depersonalization, personal accomplishment), assertiveness, stressful life events, reality shock, job satisfaction (salary), workload, overtime, social support, coping and nurse turnover were found in univariate analysis.
9	Suzuki et al. (2008) [31], Japan	Longitudinal design/ 923 RNs/2003 (June), 2003 (December), 2004 (June), 2005 (March)	Turnover between novice nurses who quit their jobs between June 2003 and March 2005/12.7%	Nurse attributes, burnout, assertiveness, stressful life events, reality shock, ward assignment preference, transfer preference, job satisfaction, social support and coping mechanisms	Cox’s proportional hazard model with the stepwise variable selection method/ 8/medium	- Support from peers and dissatisfaction with the workplace affected turnover of novice nurses. - Non-significant relationships between nurse attributes (gender, age, highest education level, living arrangements, department), burnout (emotional exhaustion and depersonalization, personal accomplishment), assertiveness, stressful life events, job satisfaction (salary), workload, overtime, social support, coping and nurse turnover were found in univariate analysis.
10	Suzuki et al. (2010) [32], Japan	Longitudinal design/ 762 RNs/2003 (December), 2004 (June)	Turnover between novice nurses who quit their jobs between December 2003 and June 2004/4.6%	Demographic attributes, burnout, assertiveness, stressful life events, reality shock, ward assignment preference, transfer preference, job satisfaction, social support and coping profiles	Multiple logistic regression analysis with the stepwise variable selection method/ 8/medium	- Hospital location being in Tokyo, burnout (physical exhaustion, emotional exhaustion and depersonalization), and dissatisfaction with the workplace affected turnover of novice nurses. - Non-significant relationships between demographic attributes (gender, age, education, ward, department), burnout (personal accomplishment), assertiveness, stressful life events, reality shock, ward assignment preference, job satisfaction (salary), workload, overtime, social support, coping and nurse turnover were found in univariate analysis.

Quality appraisal: 0–4 = LO, 5–9 = Med, 10–13 = HI, Cummings et al. [25] and Pennonek et al. [26]

*GOMS* Graduates Occupational Mobility Survey, *HMO* Health Maintenance Organization, *MBI* Maslach Burnout Inventory, *MSA* metropolitan statistical area, *NLRN* newly licensed registered nurse, *RAS* Rathus Assertiveness Schedule, *RN* registered nurse

Due to the heterogeneity of the measures of the independent variables in the included articles, a meta-analysis was not conducted. Table 3 presents the synthesis of significant findings of independent variables in the included studies. Nurse turnover variables were divided according to durations of the turnover period as follows: turnover rates for 6 months, 12 months, 24 months, and other durations. Factors were grouped as personal, work environment, nursing unit, hospital, and community factors. As mentioned above, the work environment factors were synthesized based on Price’s turnover model [16, 23]. Other factors were listed in order of similarity of the concepts. The results were categorized into significant relationships (positive, negative) and non-significant relationships.

**Table 3:** Factors affecting newly licensed nurses’ turnover

Variable (reference category)	Studies	Turnover rates for 6 months			Turnover rates for 12 months			Turnover rates for 24 months			Turnover rates for other durations		
		(+)	(−)	NS	(+)	(−)	NS	(+)	(−)	NS	(+)	(−)	NS
**I. Personal factors**													
*Demographics*													
Age	1, 3, 6^a^					3	1						6^a^
Sex (male)	1, 3, 6^a^, 7						1, 3		7				6^a^
Ethnicity (White)	1, 3						1, 3						
First language (English)	1						1						
Married (not)	1, 2^b^, 3						1, 3				2^b^		
Spousal income	1						1						
Income from other sources	1						1						
*Presence of dependents*													
Number of children living at home	1, 3						1, 3						
Children less than 6 years old	1, 3						1, 3						
*Education*													
Nursing degree (associate)	1, 3						1, 3						
Highest educational level (vocational nursing school)	8		8										
Graduation year in 2010 (2008)	7							7					
Times took NCLEX	1						1						
*Job status*													
Whether nursing was a second career	3						3						
*Health*													
Overall health	1						1						
Positive affectivity	1						1						
Negative affectivity	1						1						
Trajectories of sleep disturbance	5							5					
Unhealthy lifestyle group (discordant group)	4^c^										4^c^		
**II. Work environment factors**													
*Work attributes*													
Staff·General duty nurse (no)	1						1						
First RN job: formal orientation (no)	1						1						
First RN job: reduced workload (no)	1						1						
Hold more than one job (no)	1					1							
Income or salary	6^a^, 7									7		6^a^	
Imputed wage	1						1						
RN benefits-formal education (no)	1						1						
Total number of RN benefits	1						1						
Importance of benefits from RN job (not important)	1						1						
Months worked in current job/Organizational tenure	1, 3						1, 3						
Type of shift/Number of shifts floating	1						1						
Previous work experience-another healthcare job (no)	1						1						
Externship/Internship	1						1						
Job status (permanent)	6^a^										6^a^		
Working full time (part-time status)	1, 3				1		3						
Current position (staff nurse)	3						3						
Mandatory overtime	1						1						
Voluntary overtime	1					1							
Patient load	1						1						
Typical work schedule (day shift)	1						1						
Number of times change in supervisor	1						1						
Part of a union	1						1						
Length of commute	3						3						
*Work attitudes*													
Intent to stay	1					1							
Work motivation	1						1						
Job search behavior	1						1						
Job satisfaction	1						1						
Satisfaction													
Pay or income	6^a^												6^a^
Stability of employment	6^a^												6^a^
Potential for personal growth	6^a^												6^a^
Advancement system	6^a^												6^a^
Social reputation for the job	6^a^												6^a^
Autonomy and authority for the job	6^a^												6^a^
Work hours	6^a^											6^a^	
Social insurances and fringe benefits	6^a^											6^a^	
Physical work environment	2^b^, 6^a^											2^b^, 6^a^	
Interpersonal relationship	2^b^, 6^a^											2^b^, 6^a^	
Work content	2^b^											2^b^	
Workplace/Organization	7, 9^d^, 10		10						7			9^d^	
Profession	7								7				
Ward assignment preference	8		8										
Organizational commitment	1						1						
Organizational embeddedness	3					3							
Community embeddedness	3						3						
Organizational and community embeddedness item summed scores	3						3						
Variety	1						1						
Autonomy	1						1						
Mentor support/Supervisory support	1						1						
Support from peers	8, 9^d^		8									9^d^	
Workgroup cohesion/Collegial RN-MD relations	1						1						
Distributive justice/Procedural justice	1						1						
Promotional opportunities	1						1						
Quantitative workload	1						1						
Organizational constraints	1						1						
Work family conflict	1						1						
Family work conflict	1						1						
Job stress													
Psychological demand	4^c^												4^c^
Physical demand	4^c^												4^c^
Control	4^c^												4^c^
Boss support/Peer support	4^c^												4^c^
Physical exhaustion/Emotional exhaustion and depersonalization	10	10											
*Shocks*													
Sprains or strains	1				1								
Needle sticks/Verbal abuse	1						1						
**III. Nursing unit factor**													
Unit type	1						1						
**IV. Hospital factors**													
Magnet hospital	1						1						
Hospital size	2^b^											2^b^	
Hospital location (nonmetropolitan)	2^b^											2^b^	
Hospital location in Tokyo	10	10											
Presence of union (nonunionized)	2^b^, 7								7			2^b^	
Number of employees	6^a^												6^a^
Interaction of hospital size and salary (small, < 2.0 million won)	7								7				
**V. Community factors**													
Local job opportunity	1						1						
Non-local job opportunity	1						1						
Percent of all persons in HMOs	1						1						
Beds per 1000 population	1, 6^a^						1						6^a^
Number of hospitals in region	6^a^										6^a^		
Number of nurses per 100 beds	6^a^											6^a^	
Unemployment rate	1						1						
MSA	1						1						

HMO (Health Maintenance Organization), MD (medical doctor), MSA (metropolitan statistical area), NCLEX (National Council Licensure Examination), NS (not significant), RN (registered nurse)

Article numbers in Table 3 are according to article numbers in Table 2. Based on the findings of multivariate analysis

Variables that are conceptually similar and of the same type of relationship with turnover in the studies were grouped with slash(/)

^a^turnover rate for 48 months ^b^turnover rate for 36 months ^c^turnover rate for 6 weeks ^d^turnover rate for 21 months

## Results

### Characteristics of included studies

Ten articles were included in this review (Table 2). The year of publication ranged from 2006 to 2020. Studies originated from multiple countries, including the US [16, 27], South Korea [17, 21, 22, 28, 29], and Japan [30–32]. One article [16] was rated high with a score of 11; nine articles [17, 21, 22, 27–32] were rated medium with scores ranging from six to nine in their quality assessments. Sample sizes included in the studies ranged from 116 in Halfer [27] study to 1653 in Brewer et al. [16]. All ten studies used a longitudinal design.

A theoretical model was used in some studies. Brewer–Kovner synthesis model of direct turnover influences, which is a modified version of Price’s [23] framework was used in Brewer et al.’s [16] study, and a conceptual model consisting of four areas of turnover predictors (individual and family, nursing education, hospital characteristics, and job satisfaction), established based on previous studies was found in Cho et al.’s [21] study.

Turnover was measured by whether NLRNs left their employers between the baseline period and further data collection times. In the included studies, the measurement period ranged from 6 weeks to 48 months while the data collection time ranged from 6 months to 4 years. The mean values of turnover reported for 1 year ranged from 12 to 25% in five studies [16, 22, 27–29]; 23 to 39% for 2 years in two studies [28, 29]; 45 to 47.4% for 3 years in two studies [21, 29]; and 50 to 51.7% for 4 years in two studies [22, 29]. In Suzuki’s [30–32] studies, the turnover rate for the first and second 6 months was 4 and 4.6%, respectively, and 12.7% for 21 months. The turnover rate was not reported in one study [17].

Various contributing factors were used to examine nursing turnover, and were categorized into personal, work environment, nursing unit, hospital, and community factors (Table 3). The personal factors were sub-categorized into demographics, presence of dependents, education, job status, and health, including 19 variables. Most factors examined were work environment factors, which included 73 variables sub-categorized into work attributes, work attitudes, and shocks based on Brewer et al.’s study [16]. The nursing unit factor included the unit type. The hospital and community factors included seven and eight variables each.

Different instruments were used to measure the factors contributing to the nurses’ turnover. For example, among personal factors, Han et al. [28] measured sleep disturbance by using the General Sleep Disturbance Scale [43, 44]. In Han et al.’s [17] study, pre-employment health lifestyle variables were measured based on multiple indicators. Sleep was measured based on the recommendation of the National Sleep Foundation [36] and physical activity was measured using the Korean version of the International Physical Activity Questionnaire-Short Form [37, 38]. Alcohol consumption was measured based on the consumption frequency [39] and depressive symptoms were assessed using the Korean Center for Epidemiologic Studies-Depression scale [40, 41]. Additionally, self-rated health was measured using the item “How would you rate your usual health status in the past month?” and dichotomized responses [42]. In terms of work environment factors, job embeddedness was measured by a questionnaire [35] in Halfer’s study [27]. In Suzuki’s studies [30–32], burnout was measured by the Japanese version of the Maslach Burnout Inventory survey questionnaire [45, 46], and assertiveness was measured by the Japanese version of the Rathus Assertiveness Schedule [47, 48]. In the same studies, stressful life events were measured by Social Readjustment Rating Scale [49], and coping mechanisms were measured based on the coping taxonomy [50], although assertiveness, stressful life events, and coping mechanism were not included in the multivariate analysis performed in Suzuki’s studies [30–32].

To analyze factors contributing to the actual turnover of NLRNs, various statistical analyses were used, including binomial probit regression [16], logistic regression [27, 30, 32], Cox proportional hazards regression [17, 21, 22, 31], latent growth curve analysis [28], and survival analysis [29]. All studies used a multivariate analysis approach.

### Factors affecting NLRNs’ turnover

Based on the results of multivariate analysis, personal, work environment, nursing unit, hospital, and community factors were presented as positive significant, negative significant, and non-significant for each turnover period (Table 3).

### Relationships between factors and NLRNs’ turnover

#### Personal factors

Eight studies [16, 17, 21, 22, 27–30] examined the relationships between personal factors and NLRNs’ turnover. Nineteen personal factors were examined. As shown in Table 3, most relationships between most personal factors and NLRNs’ turnover were not significant. Only seven variables were found to have significant relationships with NLRN’s turnover. Specifically, married status (ref. unmarried) [21], the graduation year 2010 (ref. 2008) [22], trajectories of sleep disturbance [28], and unhealthy lifestyle group (ref. discordant group) [17] had a positive association with turnover. Age [27], gender (ref. male) [22], and highest educational level (ref. vocational nursing school) [30] had a negative association with turnover.

#### Work environment factors

As mentioned above, work environment factors were categorized into work attributes, work attitudes, and shock based on Price’s conceptual framework of turnover [16, 23]. The relationship between work environment factors and turnover was examined in nine studies [16, 17, 21, 22, 27, 29–32]. Most existing studies focus on work environment factors. Among the 73 work environment factors, 20 had significant relationships with turnover.

Two work attributes were positively related to NLRNs’ turnover: job status (ref. permanent) [29] and working full time (ref. part-time status) [16]. By contrast, three variables were negatively related to turnover. When nurses held more than one job [16], earned a higher income or salary [29], and worked longer voluntary overtime [16], they were less likely to leave their position. For work attitudes, only physical and emotional exhaustion and depersonalization [32] were positively related to NLRN’s turnover. That is, exhausted NLRNs are more likely to leave their position. Several variables were found to decrease NLRN’s turnover. Specifically, intent to stay [16], satisfaction with work hours, social insurances and fringe benefits [29], satisfaction with physical work environment, interpersonal relationship [21, 29], satisfaction with work content [21], satisfaction with workplace, organization [22, 31, 32], satisfaction with profession [22], ward assignment preference [30], organizational embeddedness [27], and peer support [30, 31] were negatively associated with NLRN’s turnover. Among shocks, sprains or strains [16] increased NLRN’s turnover.

#### Nursing unit factors

The relationship between a nursing unit factor and NLRNs’ turnover was examined in one study [16], and a non-significant relationship between unit type and nurse turnover was found.

#### Hospital factors

Five studies [16, 21, 22, 29, 32] investigated the relationships between hospital factors and NLRN’s turnover. Among the seven hospital factors, five had a significant relationship with turnover. When NLRNs’ worked at hospital locations in Tokyo, they reported high levels of turnover compared to hospitals in other areas [32]. The following four hospital factors were negatively related to NLRN’s turnover: hospital size and location (ref. nonmetropolitan) [21], the presence of a union (ref. nonunionized) [21, 22], and interaction of hospital size and salary (ref. small, < 2.0 million won) [22]. Magnet hospital and the number of employees were found to have no significant relationship with turnover.

#### Community factors

Only two studies [16, 29] included community factors in their multivariate analysis of the relationships with NLRNs’ turnover. Among the eight community factors, two were found to have a significant relationship with turnover [29]. When the number of hospitals in the region increased, so did the NLRN’s turnover [29]. By contrast, the number of nurses per 100 beds increased, and NLRN’s turnover decreased in the same study. Studies showed no significant relationships between the other six community factors and turnover.

## Discussion

The cost of turnover in the healthcare is considerable. Nurse turnover rates varied from 12.4% in South Korea [9] to 27.65% in the US [7]. NLRNs’ turnover is even higher (42.7%) [9]. In this review, NLRNs’ turnover was found to average 46.3% within 3 years [21], which implies that NLRNs’ are at great risk for turnover. In this review, ten articles were examined to synthesize factors affecting NLRNs’ turnover. The factors were categorized into personal, work environment, nursing unit, hospital, and community factors. Most studies examined work environment factors, except Han et al.’s studies [17, 28]. Han et al. focused on the relationship of NLRNs’ health lifestyle [17] and sleep [28] with turnover. All included studies used a longitudinal study design. Interestingly, the studies were conducted in three countries, including Japan, South Korea, and the US. In terms of datasets used in the studies, a panel survey using the Graduates Occupational Mobility Survey conducted by the Korea Employment Information Service, was employed in three studies [21, 22, 29]. Suzuki et al. [30–32] also used the same datasets with different data collection times. Han et al. [17, 28] used the same longitudinal datasets for 2 years.

Among personal factors, NLRNs’ health status was the only personal factor related to turnover. Specifically, sleep disturbance and a pre-employment unhealthy lifestyle increased NLRN’s turnover [17, 28]. Halter et al. [18] also found that stress was the strongest supported determinant of turnover at the individual level. Falatah and Salem [19] and McDermid et al. [20] did not find individual health status as a factor affecting turnover. Based on these findings, NLRNs’ health condition should be monitored and promoted. For example, sleep disturbances due to rotating shift work schedules need to be managed. Work environments that require working for long hours, overtime, and insufficient breaks are related to nurses’ adverse health outcomes [51]. Furthermore, nurses with high symptoms of sleep disturbance can be less resilient toward difficult situations, that might, ultimately, affect their turnover [52]. Unhealthy lifestyles among NLRNs can be a risk factor for turnover; therefore, managerial strategies and policy to promote healthy work environment should be developed and implemented to maintain healthy lifestyles and wellness among nurses in addition to implementing health promotion program. State policy regulating nurses’ working hours and mandatory overtime and patient load can help provide healthy work environment to prevent adverse health outcomes for NLRNs [53, 54].

In terms of work attributes, a higher income and wage for NLRNs were found to significantly reduce turnover. Appropriate compensation should be considered to prevent turnover among NLRNs. Additionally, several work attitudes - intent to stay, high levels of job satisfaction, and more peer support were negatively related to turnover. Intent to stay was a direct predictor of turnover in Price’s [23] turnover model. Job satisfaction (or dissatisfaction) was a significant factor for nursing turnover according to a previous review [18]. Thus, this review confirmed similar work environment factors that affect actual turnover among NLRNs and evidenced their significance, which should be addressed to solve high turnover among them.

An interesting finding was about peer support. Previous reviews found supervisory support and professional support [18, 19] to be strong determinants of nurse turnover. In this review, peer support was a significant factor for NLRNs. This indicates the importance of peer groups in the retention of NLRNs. Peer support opportunities were common elements of new graduate transition programs [55]. Based on this study’s finding, peer support can be actively used to prevent NLRNs’ turnover. Specifically, personal friendships and interpersonal relationships among NLRNs need to be established during their early employment period; nurse managers should promote such peer support among NLRNs [21, 32].

Regarding work attitudes among work environment factors, Suzuki et al. [32] found that physical and emotional exhaustion and depersonalization were positively related to nursing turnover. Because the process of this burnout begins long before it reaches the threshold, it is important to manage it at an early stage [32]. This type of burnout was also found to be a significant determinant of nurse turnover in a previous review [18]. During the COVID-19 pandemic, nurses’ burnout increased and was a critical issue [56]. Prevention and coping programs and strategies should be provided to NLRNs. Similarly, Brewer et al. [16] found that strains or sprains, including back injuries, were positively associated with NLRNs’ turnover. Such unexpected shock can be prevented with the use of mechanical patient-lifting devices and “no lift” policies [57]. Prevention programs and strategies for physical and psychosocial problems due to nursing work should be developed and implemented to target NLRNs’ retention.

Furthermore, adequate staffing was found to be a critical factor in reducing emotional exhaustion, injury, and job dissatisfaction [54]. Positive work environments have been emphasized to prevent aggravating nursing shortages and nurses’ well-being [54]. During the COVID-19 pandemic, more states adopted safe nurse staffing policy to provide adequate staffing and keep nurses at the bedside [58, 59]. Thus, it is important to promote and implement positive work environments with appropriate nurse staffing for NLRNs to prevent turnover among them.

In terms of hospital factors, NLRNs working in large hospitals located in metropolitan areas with unions reported lower turnover, suggesting that hospitals with more resources and support had lower turnover. By contrast, smaller hospitals located in rural areas generally have fewer resources, lower funds, difficulty retaining NLRNs, and might experience nursing shortages [60]. According to this review’s findings, small hospitals located in nonmetropolitan areas might need government and state support to retain NLRNs for providing healthcare services to the population living in remote areas. Furthermore, nursing workforce policies, at both organizational and national levels should be developed to ensure a sustainable supply of nursing workforce and to resolve geographical imbalance [21].

Factors contributing to NLRNs’ turnover are multifaceted: personal, work environment, nursing unit, hospital, and community-related. Among personal factors, health promotion and maintenance can be used to manage sleep problems and unhealthy lifestyles, which are significant factors for turnover. Prevention of occupational injuries (including strain and sprains), reduction of physical and emotional exhaustion, and depersonalization are also important to prevent NLRNs’ turnover. Peer support can be used in prevention programs for nurse turnover. Income, intent to stay, and job satisfaction should also be monitored and managed. Hospitals at risk of high turnover of NLRNs (such as small hospitals and those located in nonmetropolitan areas) might need to receive government support. Nurse staffing policy and work hour policy should be implemented and expanded. These steps can help improve the work environment, which can improve NLRN’s health and retention, reduce their turnover, and improve the quality of care and patient safety.

### Limitations

Several limitations can be found in this review. Although it attempted to include all studies investigating factors affecting NLRNs’ turnover, this review’s search strategies may have missed some studies. Particularly, the retention program for NLRNs (e.g., residency program) was not identified in this review as none of the studies investigated this factor. However, a previous review found that a residency program improved retention rates among new graduates [61]. In addition, only studies where the findings were significant may have been published in peer-reviewed journals. Therefore, when interpreting this study’s findings, we need to consider reporting bias. Furthermore, this review focused on NLRNs’ turnover in acute care hospitals. None of the studies examined NLRN turnover during and since the COVID-19 pandemic, but such studies have yet to be published. Therefore, the turnover of NLRNs in various settings may be different from our findings and needs to be investigated further.

## Conclusions

Turnover is inevitable in the process of employment over time. The complexity of turnover implies that there exists no one solution to reduce it, but work environment improvement appears to be key. In this review, ten articles were examined to synthesize factors contributing to NLRNs’ turnover in acute care hospitals. Several personal, work environment, nursing unit, hospital, and community factors were found to develop solutions that may prevent NLRNs’ turnover. Significant contributing factors of NLRNs’ turnover included: Personal factors of health status; work environment factors of physical exhaustion and emotional exhaustion, depersonalization, strains and sprains, income, intent to stay, job satisfaction, and peer support; hospital factors of hospital size, location, and unionization. Most studies focus on work environment factors, which reflects the significance of fostering healthy work conditions to prevent high turnover. These findings can be used to develop strategies and policies pertaining to the work environment, to reduce high turnover among NLRNs and support high-risk groups (e.g., small hospitals located in nonmetropolitan areas) with high levels of turnover. Further research is required to examine the turnover and retention strategies of NLRNs**.**

## Data Availability

The datasets used and/or analyzed during the current study available from the corresponding author on reasonable request.

## Abbreviations

WHO: World Health Organization
ICN: International Council of Nurses
RN: registered nurse
NLRNs: newly licensed registered nurses
